# Limb-girdle muscular dystrophies: Where next after six decades from the first proposal (Review)

**DOI:** 10.3892/mmr.2014.2048

**Published:** 2014-03-13

**Authors:** OMAR A. MAHMOOD, XIN MEI JIANG

**Affiliations:** 1 Department of Neurology, The First Hospital of Jilin University, Changchun, Jilin 130021, P.R. China; 2 Department of Neuromedicine, Mosul Medical College, Mosul 41002, Iraq

**Keywords:** LGMD, MRI, disease biomarkers

## Abstract

Limb-girdle muscular dystrophies (LGMD) are a heterogeneous group of disorders, which has led to certain investigators disputing its rationality. The mutual feature of LGMD is limb-girdle affection. Magnetic resonance imaging (MRI), perioral skin biopsies, blood-based assays, reverse-protein arrays, proteomic analyses, gene chips and next generation sequencing are the leading diagnostic techniques for LGMD and gene, cell and pharmaceutical treatments are the mainstay therapies for these genetic disorders. Recently, more highlights have been shed on disease biomarkers to follow up disease progression and to monitor therapeutic responsiveness in future trials. In this study, we review LGMD from a variety of aspects, paying specific attention to newly evolving research, with the purpose of bringing this information into the clinical setting to aid the development of novel therapeutic strategies for this hereditary disease. In conclusion, substantial progress in our ability to diagnose and treat LGMD has been made in recent decades, however enhancing our understanding of the detailed pathophysiology of LGMD may enhance our ability to improve disease outcome in subsequent years.

## 1. Introduction

Limb-girdle muscular dystrophies (LGMD) are a group of muscular dystrophies, that until the late 1980s were identified in patients by ‘diagnosis by exclusion’. Revolutionary advances in molecular biology in the last several decades have allowed the scientific community to understand and recognize this disease more clearly. Currently, there are >25 LGMD types that have been linked to specific gene loci, and they are now estimated to constitute one third of all Duchenne muscular dystrophy cases (1).

The review is constructed to cover LGMD from a variety of viewpoints and is established on the authors’ own experience and investigations, as well as inclusive MEDLINE searches on the topics of ‘limb-girdle muscular dystrophies’, ‘review’, ‘genotype-phenotype correlations’, ‘prevention’ and ‘surveillance’. In this review, we focused on peer-reviewed studies (in English) published in key scientific journals from 1995 to date, combined with several historical articles. All available articles were reviewed in depth. In consideration of the summary and usefulness for clinicians in the field of myology and for future citation, the information and references were summarized into tables.

## 2. Historical background

The term LGMD was, among hereditary muscle disorders, one of the most difficult to establish as a clinical entity. Early definitions were described by Erb where he designated a type of juvenile, scapulohumeral progressive muscular atrophy known as ‘a juvenile form of progressive muscular dystrophy’. In 1891, Erb (2) proposed to include with his cases the previous observations of Leyden (3) and Mobius (4). The theory was generally well accepted. Bell (5) was the first to differentiate this type of dystrophy from X-linked Duchenne muscular dystrophy and from autosomal dominant facioscapulohumeral muscular dystrophy. In their archetypal paper, Walton and Nattrass (6) first devised the name ‘limb-girdle muscular dystrophies’ to comprise cases of both sexes, beginning usually within the first three decades, with major involvement of scapular, pelvic girdle and trunk muscles, with sparing of facial muscles and infrequent pseudo hypertrophy, moderately severe progression and usually an autosomal recessive mode of inheritance.

With the development of physiological and histopathological means to assess muscular disorders, it rapidly seemed that a number of patients considered to be suffering from LGMD were, in fact, affected by other conditions, including spinal muscular atrophies, congenital myopathies or metabolic disorders. Hence, the clinicopathological consistency of this suggested entity was, nevertheless, again promptly disputed.

In the early development of immunostaining methods in the late 1980s, a precise distinction between LGMD and other conditions such as Becker’s dystrophy characterized by dystrophin abnormality was established (7).

In 1995, the European Neuromuscular Centre Workshop established more precise criteria for the diagnosis and classification of LGMD. More specifically, different subtypes of LGMD were grouped according to their genetic characteristics (8).

The abbreviation of the autosomal-dominant type is now LGMD1, whereas autosomal-recessive types are LGMD2. Each separate gene locus has a unique classification (Table I) (9–35).

As is evident from its long nosological history, LGMD is not an homogeneous disease. LGMD can be considered an ‘umbrella’ term under which >24 gene defects have been recognized, where single gene defects encode numerous phenotypes and vice versa.

## 3. Mechanism of action

The mechanisms of action of LGMD-involved proteins are diverse. Emphasis is moving away from the identification of structural proteins and their implications in muscular dystrophies towards investigating proteins involved in muscle fiber maintenance in response to repeated injury (dysferlin, caveolin 3 and anoctamin 5), fiber remodeling under stressful conditions (calpain 3), and post-translational modifications of proteins and the enzymes involved in these processes (POMT1, POMT2, POMTGnT1). Despite the advances in modern biochemical techniques, certain proteins still exist without defined functions (Table II) (13,35–51).

## 4. Pathophysiology

The majority of the developments in LGMD therapeutics have derived from insight gained in animal model investigations, created through the reversal of etiopathogenic agents prompting the disease or by disrupting certain steps believed to be downstream of the defect gene.

When reviewing all mutations in MYOT through the Leiden muscular dystrophy page (http://www.dmd.nl), it was evident that no null mutation has been reported to date in the MYOT gene, hence all mutations are of missense type. This evidence supports the theory that myotilin missense, but not nonsense, mutations are pathogenic in humans and there must be other proteins, such as palladin and myopalladin, which reimburse the structural and functional properties of myotilin (52). This hypothesis is further supported by the fact that the myotilin-null mouse demonstrates normal development, histology and performance, yet myotilin transgenic mice show dystrophic processes and double transgenic mice report a more severe phenotype (52,53). Authors hypothesize that genetic or pharmaceutical interference with mutant myotilin translation, may serve as promising therapeutic approaches in the treatment of LGMD. The LMNA gene encodes the lamin A/C protein, which is important in its function as a scaffold for nuclear lamina and as a vital component of cell signaling and differentiation. Currently, three pathways are implicated in the pathophysiology of LMNA-mediated cardiac and musculoskeletal dystrophies; retinoblastoma protein (pRb), mitogen activated protein kinases-extracellular signal-regulated kinases (MAPK-ERK) and transforming growth factor beta ligands (TGF-β). MAPK-ERK and pRb signaling mediate regulation of the cell cycle, while the TGF-β pathway is involved in the transduction of extracellular signals to trigger downstream TGF-β gene transcription in the nucleus (37). Several *in vivo* studies have obtained promising results by targeting these pathways. For example, Muchir *et al* despite identifying that inhibition of the ERK pathway had a minimal effect on cardiomyopathy, the impact of therapy on cardiac rhythm was not examined; the most common cause of mortality in humans (54). Further studies are essential to elucidate the precise mechanism involved in cardiac arrhythmias and skeletal muscle weakness. Myostatin or TGF-β receptor inhibition by various techniques is evolving therapeutic rationales for caveolin 3 mutations. Yet, these treatments were applied to a particular mutation (55). Whether these therapies are effective on other types of mutations or other models remains to be explored.

Accumulating lines of evidence have demonstrated that gene transfer of CAPN3 and mutated myostatin propeptide delivery were efficient therapies in investigations utilizing the calpainopathy murine animal model. However in this study, the duration for gene persistence in muscles was relatively short (9 days), the mode of gene delivery (intramuscular) was not sufficient to improve lifestyle, and the parameters used for assessing improvement were poor, such as the contractile force, which was measured *ex vivo* (56). Several novel therapeutic strategies have been piloted to treat DYSF gene (DYSF) deficiency. The most well characterized study was that performed by Han *et al*, who disrupted the complement component C3 (C3), that is considered to be a crucial mediator of inflammatory cascades in DYSF deficient mice (57). Nonetheless, several queries were raised against the proposed model, including the limitation that the contractile force was not examined. In addition, the authors hypothesized that it is complement activation rather than contraction-induced injuries that is responsible for muscle weakness in the DYSF deficient mouse, however some dysferlinopathic patients have no infiltrates on their biopsies (58). It is unlikely that membrane attack complexes (MAC) underlie muscle weakness, given that the same study identified that C5 ablation (a terminal component of the MAC pathway) had minimal effects. This issue should be addressed in future trials when utilizing inflammatory and non-inflammatory models. Whether complement C3 inhibitors (easily administered and more convenient for human trials) will have a similar effect as C3 ablation remains to be clarified. In sarcoglycanopthies, various therapeutic stratgies have been investigated *in vitro* and *in vivo*, including gene transfer, stem cell grafting, myostatin blockade and calcium channel blockers. Notably, sarcoglycanopathies conveyed more success of gene therapy than other LGMD subtypes due to small size genes. Lastly, calpain 3 inhibition provided a rationale for the treatment of titinopathy.

Though numerous therapies have proved efficacious and safe in several different murine models, caution should be exercised when considering the applicability of these treatments to humans, given the evident differences in size, life span, genetic variants and immunological complexity between the two species. Table III (54–57,59–82) summarizes the majority of murine models constructed and the interventions applied with their results.

## 5. Disease markers

With recent advances in therapeutic trials, more light has been shed on the clinical outcome measures that can be utilized to predict disease activity, regression and treatment efficacy without requiring multiple muscle biopsies.

There are multiple disease markers for LGMD, some of which are well known, and others which have only been identified in recent years. For example, high creatine kinase (CK) levels indicate disease activity; while low levels may indicate fibrosis or therapeutic responsiveness. In cases of local delivery of target therapy, CK level is not helpful. Hand grip strength, Medical Research Council (MRC) scale, Gardner-Medwin and Walton scales or other clinical scales are beneficial for assessing clinical outcome, however inter and intra examiner variability and false-negative results in depressed patients reduces the efficacy and reliability of this method. Another measure utilizes contrast agent-enhanced MRI scanning. Typically, albumin-targeted contrast agent, (MS-325) does not enter into myocytes. In membranopathies, the dye is usually observed in the sarcoplasm. The technique is considered a robust noninvasive disease follow-up tool in the therapeutic trials of the sarcoglycanopathies (83). Measuring the secreted alkaline phosphatase levels (Se AP) in blood (succeeded in murine models), involves insertion of the secreted alkaline phosphatase gene with the gene of interest into a viral vector. Following this, simple blood-based assay of Se AP can predict gene expression in specific muscles. The assay overcomes limitations of CK level identification that is nonspecific and minimally affected in cases of local delivery of the gene (84). Another approach measures dysferlin and calpain 3 expression levels using circulating monocytes that reflect expression level sin muscles given that the target therapy is delivered systemically (61).

### Luciferase assay

Luciferase assay is considered as ‘regeneration reporter’ and its level mirrors the number of centrally nucleated fibers and embryonic heavy chain myosin positive cells (61).

### Neutralizing antibodies and interferon (INF)-γ to r AAV

Measuring antibody titer or mononuclear cell mediated INF-γ secretion, by enzyme linked immunosorbent assay (ELISA) and enzyme-linked immunsorboent spot (ELI Spot) assays, respectively, were used to detect degree of immune rejection to inserted vector gene (85).

## 6. Disease prevalence

**Figure 1:**
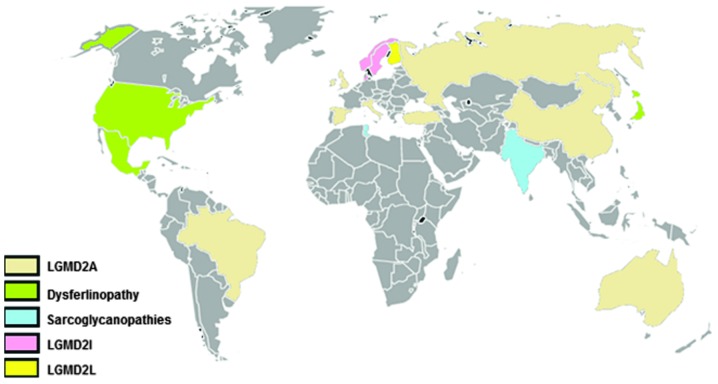
World map with the most common LGMD forms represented in colors. Grey color indicates countries with no known cohort. In Spain, Italy, England, Turkey, Russia, China, Brazil and Australia, the most common type was calpainopathy. LGMD2I was more frequent than other forms in the Scandinavian Peninsula. However, dysferlinopathy was the most frequent in US, Japan and Mexico. In India, sarcoglycanopathies had the highest incidence, whereas in Finland, anoctaminopathy ranked the first (25%) amongst other forms.

With the exception of countries such as Norway, Denmark and Finland, where the founder effect was determined, LGMD2A is the most frequent type of LGMD worldwide, followed either by dysferlinopathies in some areas or by sarcoglycanopthies in others (Fig. 1). The relative frequencies of the subtypes that exist among various ethnicities are described in Table IV (86,87,89–92,94–107). LGMD is considered the second most common muscular dystrophy in England, Mexico and Turkey, after dystrophinopathies, with a disease prevalence of up to 1/14,500 and a carrier frequency of up to 1/150 (86–88).

## 7. Genotype-phenotype correlation

Clinical presentations of LGMD disorders vary among patients of the same subtype, or even within the same family (108). Genotype-phenotype correlational analysis is however, difficult to predict and it represents one of the most challenging obstacles in the field of genetic disorders. Trials to elucidate the specific clinical picture for individual genetic subtypes were futile (108).

Currently, two null mutations in the CAPN3 gene have been associated with severe phenotype, early onset and risk of being crippled (90). Natural exon 32 skipping of the DYSF gene notably reduced the severity of symptoms in a mother of two severely affected daughters by homozygous mutation (109). Mild features are most commonly reported in relation to the common Asian mutation c.2997G>T, p.W999C). However, the mutation has also been described in association with a variety of phenotypes (110–112). In cases of LGMD2H, it has been identified that mutations gathered in the NHL (named after the proteins NCL1, HT2A and LIN-41) domain result in LGMD2H/sarcotubular myopathy, whereas in Bardet-Biedl Syndrome, mutations are most commonly located in the B-box region of the gene. This may suggest that mutations in the NHL area render individuals more susceptible to muscular disorders (113). Late disease onset, mild phenotype, less susceptibility to loss of ambulation and more liability to myoglobinuria, are consistent features observed in patients homozygous to c.826C>A (p.L276I) of the FKRP gene compared with patients heterozygous to the same mutation (96). While several LGMD forms are phenotypically heterogeneous, it appears that ‘hot spot’ c.191dupA mutation in the ANO5 gene is associated with a more homogeneous phenotype (94). Of note, no LGMD2M patients to date have exhibited a 3 kb retrotransposal insertion in the FKTN gene, a founder mutation accounted for 87% of Fukuyama type congenital muscular dystrophy (114). Finally, it has been identified that mutations clustered in immunoglobulin-like fold and coil 2 of the LMNA gene are inconsistently correlated with the autosomal dominant form, LGMD1B (115).

Matching gene expression profiles of normal and affected muscles, identifying the crystalline structure of the protein of interest and recognizing the precise function of each protein domain, are approaches that will improve our understanding of the associations between various pathogenic mutations and disease presentation in LGMD (116).

## 8. Diagnostic strategy

Clinical, electrophysiological, imaging, biochemical and genetic testing techniques collectively should be utilized and tailored according to specific LGMD patient cases. Not only will this facilitate diagnosis and provide individualized genetic counseling to proband and relatives, but will also enhance the understanding of the underlying pathophysiology, to allow delivery of a therapeutic strategy that targets the precise pathways that are specific to that patient.

### Clinical

Historical analysis and clinical examination are commonly used approaches to identify specific LGMD subtypes.

### Age of onset

The vast majority of autosomal recessive LGMD cases have teenage onset-progressive muscle weakness, however, there are exceptions to this rule. In certain dystroglycanopathies, sporadic dysferlinopathy cases may start exhibiting perinatal ‘floppiness’ and mild weakness as late as 70 years-old (117,118). While calpainopathy and dysferlinopathies tend to manifest in late childhood to late teens, early childhood onset indicates the diagnosis will be a sarcoglycanopathy or dystroglycanopathy. The majority of, but not all, autosomal dominant LGMD patients begin experiencing symptoms following their twenties.

### Pattern of distribution of muscles weakness

LGMD is associated with marked clinical disparity. As the rule of thumb, a defect in membrane scaffolds cause predominant proximal myopathies, whereas sarcomeric protein deficiencies typically result in initial distal myopathies. The exceptions to this include distal Miyoshi myopathies (MMs), which are associated with the membrane patch proteins dysferlinopathy and anoctaminopathy. Scapulohumeral muscle weakness or ‘Erb phenotype’ is most commonly observed in sarcoglycanopathies, calpainopathies and Duchene’s type of LGMD2I, while pelvic muscle weakness or ‘Leyden-Mobius phenotype’ (LM) is the leading indicator for the majority of LGMD subtypes. While calf, thigh and tongue hypertrophies are mostly encountered in sarcoglycanopthies and dystroglycanopathies, deltoid hypertrophy appears to be restricted to dysferlinopahies (119,120,121).

### Skeletal manifestations

Contracture is reported in numerous types of LGMD, however predilection is given to laminopathy, calpainopathy, dystroglycanopathies and anoctminopathy. While kyphoscoliosis and lordosis manifest in calpainopathies, dystroglycanopathies and plectinopathies, features such as bent and rigid spines, appear to be limited to dysferlinopathies (112,122).

### LGMD and heart

With the exception of myotilinopathy, laminopathies, telethoninopathy and LGMD2I, cardiac muscles are spared in the majority of LGMD subtypes or seldom involved in others (LGMD2A, 2B and 2C-2F). Patients with LGMD may present with wide range of cardiac abnormalities e.g., atrial fibrillations, flutters, atrio-ventricular conduction blocks, supraventricular, ventricular ectopic beats, ventricular tachycardia and sudden death commonly seen in the laminopathy patients; on the other side, dilated, hypertrophic and restrictive cardiomyopathy are noticed in some sarcoglycanopathies and a third of LGMD2I cases. Cardiac problems may precede, overlap with or follow skeletal muscle weakness. Periodical cardiac monitoring and pacemaker or defibrillator implantation are warranted in certain cases (123).

### LGMD and pulmonary function

Respiratory muscle weakness is a rare manifestation and predominantly indicates diagnosis of LGMD2I, myotilinopathy and occasionally, LGMD2A and LGMD2C-2F. Patients with LGMD2I may also develop respiratory failure while ambulant. Pulmonary function tests should be part of routine examination in suspected LGMD subtypes.

### Extra muscular features

Detailed neurological exams often provide an implication of the specific LGMD form. While neuropathy, paresthesia, nasal speech and dysarthria appear to commonly occur in myotilinopathies; opthalmoparesis, foot drop, and paresthesias have been sporadically reported in LGMD1C, 2G and 2H respectively (124,125). In dystroglycanopathies, with the exception of LGMD2I, muscle weakness is usually coupled with cognitive impairment, which has serious psychosocial consequences for the patient and their family. Patients with LGMD2I usually have normal intellect, nonetheless there may be some difficulties in visiospatial planning and memory function (126).

### Clinical course

**Figure 2:**
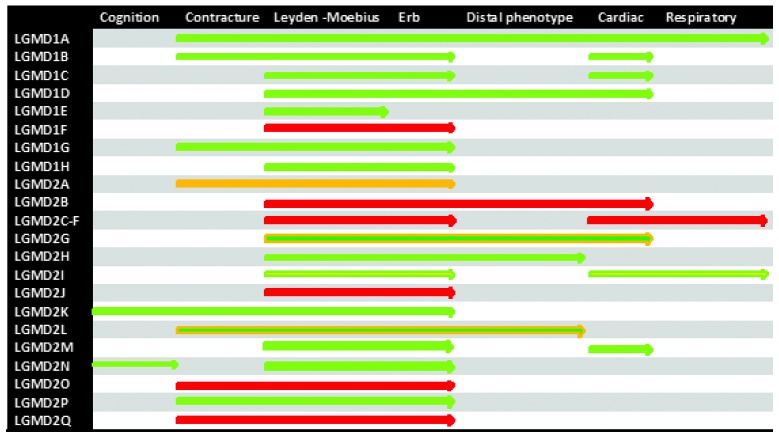
Disease spectrum for rapid recall of LGMD subtypes. Note: green color represents slow progression, yellow color represents moderate progression, red color represents rapid progression and mixed color represents variable progression depending upon either type of the mutation or gender factor (see text).

LGMD subtypes have a characteristically slow progression, with weakness most commonly beginning in the proximal lower limbs and sometimes the distal limbs. This is followed by weakness in all other limbs and then permanent disability is established two to three decades after disease onset. The subtypes LGMD1D, LGMD2L and LGMD2M represent slowly progressive diseases and patients are mildly affected and remain ambulant (94). Rapid progression is a classical feature of dysferlinopathies, sarcoglycanopathies and plectinopathies. In some LGMD forms there may be inconsistencies in the progression of the disease between male and female-affected patients. In female mild-onset patients, estrogen is considered to impact disease progression. LGMD2G and LGMD2L are examples of LGMD groups with inconsistent disease progression between male and female patients. The study discerned another type of LGMD that is also characterized by a two-peak onset; rapid progressive Duchene like childhood onset and milder Becker-like adolescent to adulthood onset. LGMD2I is representative of this set (96). Fig. 2 denotes disease spectrum for the rapid recall of clinical features of LGMD subtypes.

### Clinical constellations

LGMD remains an expanding group of diseases, and approximately one third of cases have not yet been associated with a specific subtype. Certain clinical constellations often favor specific LGMD subtypes, and eliminate a wide array of unnecessary genetic tests. In patients presenting with distal muscular weakness, contractures and cardiac problems in adulthood, myotilinopathy should be considered. Limb-girdle muscle weakness with a family history of sudden death and late onset contractures, most commonly indicate a laminopathy, whereas myalgia, rippling phenomenon, in association with proximal limb muscle weakness in the first four decades of life should raise suspicion of caveolinopathy. Proximal limb muscle weakness with contractures and calf atrophy in teens are features consistently associated with LGMD2A. Early childhood onset muscle weakness with cognitive impairment is a pathognomonic feature of dystroglycanopathies. In patients with cardiopulmonary involvement in association with myopathy and myoglobinuria, LGMD2I should be suspected.

### Biochemical, imaging and electrophysiological studies

#### Muscle enzymes (CK)

CK levels are either normal or mildly elevated in the majority of autosomal dominant LGMDs, whereas in autosomal recessive types, they are highly elevated. While high CK levels in dysferlinopathies, dystroglycanopathies and sarcoglycanopathies are usually correlated with disease activity, calpain 3 deficient patients typically manifest mild to moderate CK level elevation. However, normal and high levels of CK have been sporadically described amongst the disease subtypes. Although CK levels are not specific measurements for the diagnosis of muscular diseases, they often act as a useful tool for guiding physicians to a specific disease process and exclude metabolic or acquired myopathic diseases (127).

#### Magnetic resonance imaging (MRI)

Novel diagnostic tools have been recently introduced to the field of neuromuscular disorders. Studies have utilized modern imaging facilities to facilitate in the diagnosis of highly complicated genetic diseases and to allow physicians to direct patients for further protein and gene testing. The distinction between the most common subtypes (LGMD2A and LGMD2I) that account for up to 80% of recessive LGMD disorders (in some ethnic groups) is now possible using imaging techniques (128). LGMD2A is characterized by the involvement of gluteus maximus, the posteromedial thigh area and the selective involvement of medial calf muscles. This is in marked contrast to LGMD2I where calf muscles are non-selectively involved. In addition, winging of scapulae and calf atrophy are more evident in calpainopathies than in LGMD2I. The anterior thigh muscles are more affected than the posterior ones in α-sarcoglycanopathy, where the calf muscles are relatively spared. This is in contrast to dystrophinopathies, where early and striking changes in the gastrocnemii muscles are prominent (128,129). The specific pattern of affected muscles was eventually delineated in anoctaminopathy, as demonstrated by high signal intensities in posterior thigh muscles, partial atrophy of quadriceps and posteromedial calf affection (89). In myotilinopathy, distal muscle groups represented by the calves are more affected than proximal peroneal muscles and medial pelvic, thigh and lower leg muscles are more involved than lateral sets. The opposite is true for desminopathy (130).

#### Electrophysiological studies; nerve conduction velocity (NCV) study and electromyography (EMG)

Electrophysiological studies are highly important in differentiating myopathic diseases from neurogenic ones, particularly if the patient presents with distal myopathy or contracture deformity where other differential diagnoses, including Charcot-Marie-Tooth syndrome, may be considered. However, neurogenic damages have been sporadically reported in calpainopathy (131) and dysferlinopathy (unpublished observations).

#### Muscle biopsy: optical and electron microscopy (EM)

Muscle biopsy is a transitional step in the diagnostic algorithm of muscle disorders. Several studies are ongoing to simplify the diagnosis of muscular disorders using more superficial tissues like skin (132).

A wide spectrum of optical microscopic alterations, ranging from mild to severe degenerative muscle changes, has been described in LGMD biopsy specimens. Aside from the myopathic features (fiber size variation, internal myonuclei and fiber splitting), inflammatory components appear frequently in biopsy analysis of specific LGMD subtypes like dysferlinopathies (133), anoctaminopathies (94) and dystroglycanopathies and rarely in others, including LGMD2A (134), LGMD2C (135) and LGMD2D (manuscript in press). While rimmed and non-rimmed vacuoles are non-classical features in LGMD disorders (observed mainly in autosomal dominant patients), amyloid deposits are frequently encountered in dysferlinopathies and anoctaminopathies. Features of chronicity, including lobulated fibers, cytochrome C oxidase (COX) negative fibers and fibers with focal areas of reduced or absent nicotinamide adenine dinucleotide tetrazolium reductase (NADH-TR) are frequently detected in calpainopathy. Desminopathy should be considered in cases with menadione-linked nitro blue tetrazolium (M-NTB) positive cytoplasmic inclusions (12). In cases with only trivial muscle pathology, biopsy analyses are usually non-specific or even normal. However, many genetically confirmed LGMD cases have been mistakably diagnosed as acquired myopathies (136). Hence, EM and immunohistochemistry are essential to establish the diagnosis of LGMD.

EM has a crucial role in diagnostic analyses of certain LGMD subtypes particularly those associataed with myofibrillar proteinopathies. Cytoplasmic filamentous inclusions, spheroid bodies, myofibrillar protein aggregates and Z-disc streaming are features that are commonly diagnosed as myotilinopathies, whereas excess subsarcolemmal granulofilamentous material is in keeping with the characteristics of desminopathy (137). With the exception of laminopathy and myotilinopathies, myonuclei are usually spared in LGMD, which is a feature that facilitates its distinction from sporadic and hereditary inclusion body myopathy. Recently, EMs has been used to explore etiopathogenic mechanism underlying some dystrophic processes. These studies identified plasma membrane defects, basal lamina duplications and submembranous flocculations detected in LGMD2B and LGMD2L as the key indicators of a membrane reseal defect. Of note, some features e.g., vacuolar structures, dilated T tubules and myelin bodies, are non-specific and are shared with a variety of muscular disorders.

#### Immunohistochemistry (IHC) and western blot (WB) assays

With respect to their diagnostic significance, WB and immunostaining of muscle sections with antibodies against dysferlin (138), sarcoglycans (139), caveolin 3 (140) and telethonin (141) are now the ‘gold standards’ owing to their high specificities and cost-effectiveness.

In LGMD2A, this approach is often hindered by incomplete sensitivity and specificity. The process of staining muscle sections with available antibodies against calpain 3 is generally disputed as different staining patterns have been detected in abnormal LGMD2A biopsies; however, the specificity of western blotting has been improved by assessing calpain 3 autolytic activity (142,143). In addition, quantitative analyses of calpain 3 bands offer high diagnostic yield ranging from 84% in certain studies to 100% in others (144–147).

Other specific antibodies are those raised against the C-terminus of the giant titin protein and against truncated TRIM32 protein due to compound heterozygous mutations (148,149). A putative broken linkage between the sarcolemma and sarcomere, due to plectin 1f isoform mutation, most commonly results in absent sarcolemma immunostaining that is highly suggestive of plectinopathy. Whereas, reduced expression of dystroglycan-α and laminin-α2 overlay is a sensitive diagnostic indicator for dystroglycanopathies. However, this approach should be interpreted in association with clinical data, to facilitate selecting a specific gene testing method for the patient.

Proper sample handling, freezing and homogenization usually solve the limitation of denatured proteins, when certain antibodies cannot identify and may not be appropriate for biochemical assays. On the other hand, the masking effect of the epitope of an antibody may provide an inaccurate signal and some cases can be easily be overlooked (150).

High expression of calpain 3 and dysferlin in monocytes and the skin has reduced the necessity for muscle biopsies (132,151). Furthermore, multiplex blot analyses technique allows the investigator to envisage protein interaction and secondary reductions more clearly. Also, ‘reverse protein array’ confers high sensitivity to minimal protein changes that make it suitable to follow-up markers and predict drug responsiveness in upcoming trials (152).

#### Genetic diagnosis

Careful analysis of clinical and pathological findings and physiological and biochemical data, often provides crucial clues for the diagnosis of a distinct LGMD form. Currently, the documentation of a pathogenic mutation is currently warranted as a tool for identifying the diagnosis of a hereditary muscular disorder.

Genetic analyses are presumed to offer diagnosis for ~99% of cases with known gene loci. However in some types, such as LGMD 2A, virtually 25% of cases have no defects in the CAPN3 gene and ~22% have only one affected allele (153). It was estimated ~10–15 % of mutations are either intronic or subtle exonic splice sites. Therefore, muscle flesh, skin or in special cases (LGMD2A, 2B) blood monocytes are necessary to obtain mRNA (153,154). With the exception of selected centers in USA and certain European countries that offer genetic analyses for LGMD patients, genetic diagnosis is only affordable on a research basis. Nonetheless, the common hot spot mutations in certain ethnicities can be targeted prior to running whole gene sequencing (Table V) (13,14,25,27,35,90,92, 94,95,96,101,104,141,148,155–162).

As delineated in this review, LGMD is still an underdiagnosed entity and families with no identifiable gene locus may benefit from approaches like linkage analysis (34). Furthermore, gene chips, exome and wide genome sequencing are promising diagnostic tools for the diagnosis of *de novo* mutations, yet due to the high cost and extensive number of sequence variants, they are limited by challenging monetary and interpretation tasks (163).

## 9. Prevention and surveillance

The optimum time for determination of genetic risk, elucidation of carrier status, and discussion of the availability of prenatal testing, is prior to pregnancy. It is appropriate and necessary to offer genetic counseling to young adults who are affected, are carriers, or are at risk of being carriers. The identification of certain LGMD subtypes has led to changed advice e.g., certain sarcoglycanopathies (autosomal recessive trait) had previously been diagnosed as Becker muscular dystrophy (X-linked recessive trait that mainly transmitted to males) (164).

In certain LGMD disorders, joint contracture, cardiac or respiratory muscle dysfunctions arise earlier and later in the disease course. Regular surveillance with early physiotherapy, orthotics and stretching exercises facilitate joint deformities and delays disabilities for approximately two years (165). Consistent monitoring of cardiac function with early pacemaker or improved implantable cardioverter-defibrillator (ICD) instruments, may rescue the lives of certain laminopathy patients. Consistent monitoring of respiratory function, using of annual influenza vaccines, early physiotherapy, nocturnal ventilation with use of mucolytic and antibiotics if necessary, are all strategies that will reduce the rate of hospitalization and delay the need for tracheostomy and mechanical ventilation for several years. The majority of LGMD patients suffer from depression, social isolation, low self-esteem and culpability (166), so often pyschiatric therapy is of much benefit too.

The patient may be monitored for cardiac and respiratory function in the outpatient neurology clinic, nonetheless, a multi-disciplinary team is recommended to improve outcome and to confirm the optimal timing for intervention. Finally, in cases with unidentified gene defects, should they develop cardiopulmonary or skeletal complications then the above mentioned principles will apply.

## 10. Management

Like other hereditary disorders, despite extensive research, there are currently no therapeutic strategies to treat LGMD. The existing management techniques include emotion and physical support, such as in the use of canes, walkers, splinters, surgical intervention in case of contracture deformities, and support of cardiac and respiratory functions in cases of myotilinopathy, laminopathy and dystroglycanopathy.

Several clinical trials have been completed in humans and others are actively recruiting. These trials in humans have been initiated due to the trials in murine models which successfully demonstrated gene, cell transfer and pharmaceutical therapy in prinicipal (54–82).

While ‘replacement therapy’ of the defect using gene- or cell-based therapy is the gold standard, pharmaceutical therapy seems to be a more favorable approach, due to the fact the medicine can be evenly distributed to the whole body and is suitable for both modes of inheritance. The proposed pharmaceutical approaches act by targeting specific pathophysiological pathways in the disease. Increasing muscle mass by enhancing positive regulators or by inhibition of negative regulators of muscle growth, such as neutralizing myostatin antibodies, have been utilized in clincal trials, however unfortunately, despite proving safe and tolerable, demonstrated negative results at the endpoints (167). Another therapeutic target is calcium channels, which are more permeable in sarcoglycanopthies, caveolinopathies and other LGMD forms. These observations have been confirmed by reports that anecdotal improvement of CK level was demonstrated in an MM Japanese patient treated with dantrolene to reduce muscle pain, which is a drug that can block calcium channels. Furthermore, a combination of lisinopril (a calcium channel blocker agent) and Co Q10 are included in the upcoming trials in the treatment of LGMD. In dysferlinopathies, treatment startegies differ, because inflammatory mechanisms are often active in the DYSF mutant muscles. These approaches include, the use monoclonal antibodies like rituximab to block B cell activation or the use of intravenous immunoglobulin to prevent complement attack complex activations. Recently, vitamin D3 has been shown to possess DYSF promoter properties and has improved dysferlin expression in muscles and monocytes of DYSF mutation carriers (Table VI) (30, 85,167–174). The beneficial effect of steroids in the treatment of sarcoglycanopthies and dystroglycanopathies is a matter of interest, but the mechanism underlying how they improve muscle strength remains elusive. Similarly, this also applies to creatine monohydrate and Co Q10. Another encouraging potential strategy involves the use of calpain inhibitors to stop ubiquitous degradation of misfolded proteins in the Golgi apparatus, however there is no evidence of their effect in humans. Lastly, identifying drugs that upregulate surrogate proteins like ɛ-sarcoglycan in cases of α-sarcoglycanopthies, Integrin α7β1 in cases of γ-sarcoglycanopathies, or LARGE in cases of dystroglycanopathies, may also be of potential interest.

## 11. Conclusion

In recent years, our understanding of LGMD has advanced, with regards to disease occurrence, founder effect in some locations, certain aspects of pathophysiology and phenotype-genotype correlations. In addition, elucidation of hot spot mutations, disease biomarkers, general strategies of the diagnosis and treatments would point toward efficient and safe follow up, and intervention. However, more emphasis should be placed on pathogenesis, updating of diagnostic guidelines, with regular assessment and close follow up of disease progression to better elucidate the history of the disease and to enrich translational research. Universal patient registry and collaborated multicenter LGMD disease projects should be encouraged, to provide invaluable insight into its common and unique features and to settle standardized patient care.

## 12. Future perspectives

In the past six decades, advances in the field of molecular biology has opened new avenues to understand the LGMD clinical diagnosis, classification, pathogenesis and treatment possibilities. However, our understanding of the pathophysiology of the majority of LGMD forms is still in its infancy.

The majority of of autosomal dominant disorder mutations behave in a ‘dominant negative fashion’. It remains unresolved why single amino acid substitution results in negative adverse function. What is equally intriguing, is whether all mutations act by same mechanism and whether protein interaction with other known and yet undisclosed proteins affect the phenotypes of these diseases. Multiple strategies have been devised to overcome dominant negative cytotoxicity in animal models, including RNA interference, however their safety and efficacy needs to be proved in forthcoming clinical trials.

While the pathogenicity of most autosomal recessive disorders remains to be elucidated, a combination of biochemical tests and the availability of variable animal models have provided invaluable clues for physiological functions of key proteins involved in LGMD. However, it remains unclear how protein deficiencies result in muscle fiber degeneration. A parallel question is why some ubiquitous proteins are involved specifically in muscle diseases. What is equally unclear, is how congenital and adult muscular diseases are produced by the same mutation. The use of a variety of animal models with different types of mutations within the same gene and observing their effects may feasibly solve the ‘paradox of single gene and multiple phenotypes’.

## Figures and Tables

**Table I:** LGMD classification.

Form	Locus	Gene	Proteinopathies	Key references
Autosomal dominant				
LGMD1A	5q31	*MYOTM*	Myotilinopathies	(9)
LGMD1B	1q11-q21	*LMNA*	Lamin A/C opathies	(10)
LGMD1C	3p25	*CAV3*	Caveolinopathies	(11)
LGMD1D	2q35	*DES*	Desminopathies	(12)
LGMD1E	7q36	*DNAJB6*	HSP40/DNAJ	(13,14)
LGMD1F	7q32.1-q32.2	-	-	(15)
LGMD1G	4p21	-	-	(16)
LGMD1H	3p23-p25	-	-	(17)
Autosomal recessive				
LGMD2A	15q15.1	*CAPN3*	Calpainopathy	(18)
LGMD2B	2p13	*DYSF*	Dysferlinopathies	(19)
LGMD2C^a^	13q12	*SGCG*	γ-sarcoglycanopathy	(20)
LGMD2D^a^	17q12-q21.33	*SGCA*	α-sarcoglycanopathy	(21)
LGMD2E^a^	4q12	*SGCB*	β-sarcoglycanopathy	(22)
LGMD2F^a^	5q33	*SGCD*	δ-sarcoglycanopathy	(23)
LGMD2G	17q12	*TCAP*	Telethoninopathy	(24)
LGMD2H	9q31-q34	*TRIM32*	E3-ubiquitin ligase	(25)
LGMD2I^b^	19q13	*FKRP*	Fukutin-related protein	(26)
LGMD2J	2q31	*TTN*	Titinopathies	(27)
LGMD2K^b^	9q34.1	*POMT1*	POMT1	(28)
LGMD2L	11p14.3	*ANO5*	Anoctaminopathies	(29)
LGMD2M^b^	9p3	*FKTN*	Fukutinopathies	(30)
LGMD2N^b^	14q10-q24	*POMT2*	POMT2	(31)
LGMD2O^b^	1p34-33	*POMGnT1*	POMGnT1	(32)
LGMD2P^b^	3p21	*DAG1*	Dystroglycan	(33,34)
LGMD2Q	8q24.3	*PLEC*	Plectinopathies	(35)

Nomenclature of LGMD1D/1E was according to OMIM.

a Sarcoglycanopathies;

b dystroglycanopathies.

LGMD, limb-girdle muscular dystrophies.

**Table II:** LGMD subtype: Proteins and putative functions.

Form	Protein	Site	Anticipated function (ref.)
LGMD1A	Myotilin	Sarcomere	Z-disc structure protection, anchorage of thin filaments to the Z-disc (36)
LGMD1B	Lamin A/C	Nuclear membrane	Nuclear membrane stabilization, cell signaling, differentiation (37)
LGMD1C	Caveolin 3	Sarcolemma	Membrane trafficking, signal transduction (38)
LGMD1D	Desmin	Sarcomere	Assembly and the formation of the extra-sarcomeric cytoskeleton (39)
LGMD1E	HSP40/DNAJ	Ubiquitous	Protecting client proteins from irreversible aggregation (13)
LGMD1F	-	-	
LGMD1G	-	-	
LGMD1H	-	-	
LGMD2A	Calpain3	Cytosol, sarcomere	Sarcomeric remodeling; zygomatic and structural function (40,41)
LGMD2B	Dysferlin	Sarcolemma	Membrane repair and vesicle trafficking (42)
LGMD2C	γ-sarcoglycan	Sarcolemma	Part of DGC, involved in membrane integrity, cell signaling (43)
LGMD2D	α-sarcoglycan	Sarcolemma	Part of DGC, involved in membrane integrity, cell signaling
LGMD2E	β-sarcoglycan	Sarcolemma	Part of DGC, involved in membrane integrity, cell signaling
LGMD2F	δ-sarcoglycan	Sarcolemma	Part of DGC, involved in membrane integrity, cell signaling
LGMD2G	Telethonin	Sarcomere	Sarcomeric assembly, titin anchor (44)
LGMD2H	E3-ubiquitin ligase	Cytosol	Involved in ubiquitin-proteasome pathway (45)
LGMD2I	Fukutin-related protein	Extracellular	Unknown, glycosylation of α-dystroglycan (46)
LGMD2J	Titin	Sarcomere	Sarcomeric scaffold, elasticity, force bearing mechanism, cell signaling (47)
LGMD2K	POMT1	Extracellular	Catalyze the first step in O-mannosylation of α-DG (48)
LGMD2L	Anoctamin 5	Sarcolemma	Calcium-activated chloride channel function, reseal mechanism (29)
LGMD2M	Fukutin	Extracellular	Unknown, putative phospholigand transferase (49)
LGMD2N	POMT2	Extracellular	Catalyze the first step in O-mannosylation of α-DG (48)
LGMD2O	POMGnT1	Extracellular	Catalyze the second step in O-mannosylation of α-DG (50)
LGMD2P	Dystroglycan	Sarcolemma	Connect extracellular medium to intracellular scaffold (51)
LGMD2Q	Plectin	Sarcomere	Cytoskeleton system linker (35)

LGMD, limb-girdle muscular dystrophies; ref, reference; DGC, dystrophin glycoprotein complex.

**Table III:** Murine models with assumed therapy.

LGMD	Animal model	Description	Comment (ref.)	Intervention (ref.)	Result (ref.)
LGMD1A	Myo^−/−^	Knockout mouse	Normal		
	Myo^+/T57I^	Transgenic mouse	Phenotype similar to myotilinopathy		
	DTg	Double transgenic	More severe phenotype than TgT57I, Tg WT		
LGMD1B	Lmna^H222P/H222P^	Knockin mouse	More relevant to human laminopathy	ERK inhibition (PD98059) (54)	Reverse DCM in mice
	Lmna^−/−^	Knockout mouse	Less relevant to human laminopathy		
LGMD1C	Cav-3^−/−^	Knockout mouse	Muscle disease, HCM, defect in T-tubules		
	Cav-3^+/P104L^	Transgenic mouse		Myostatin (M) inhibition (55)	Revere atrophy, weakness
				TGF-β receptor inhibitor (59)	Revere atrophy, weakness
LGMD2A	Capn3^C129S/C129S^	Knockin mouse	Proteolytically inactive, structurally intact	AAV delivery mutated (M) (56)	Revese atrophy, weakness
	Capn3^−/−^	Knockout mouse	No calpain-3	AAV delivery calpain 3 (60)	Revese atrophy, weakness
	Capn3 Tg	Transgenic mouse	Normal		
LGMD2B	A/J	Spontaneous	Retrotransposon insertion in intron 4	Diltiazem (61), pyridostigmine (62)	Improved contractile function
	B6.A/J	By breeding	Retrotransposon insertion in intron 4	Dual AAV gene transfer (63)	Clinical, biochemical imp.
	SJL/J	Spontaneous	Splice site mutation at exon 45	HUCB i.v. administration (64)	Biochemical imp.
				Q10 and resveratrol (65)	Histological imp
	B10.SJL	By breeding	Splice site mutation at exon 45		
	Dysf^−/−^	Knockout mouse	Deletion of exon 45	Genetic disruption of C3 (57)	Improved muscle pathology
LGMD2C	gsg^−/−^	Knockout mouse	Exon 2 disruption	AAV gene transfer (66)	Biochemical imp.
				Myostatin blockade (67)	Improve function not histology
	gxi	Double knockout	Lacking both integrin α7 and γ-sarcoglycan	Integrin α7β1 (68)	Compensate γ-sarcoglycan
LGMD2D	Sgca^−/−^	Knockout mouse		AAV gene transfer (69)	Biochemical imp.
				Mesoangioblasts i.a. (70)	Biochemical imp.
				Deacetylase inhibitors (71)	Reverse morphology, function
	SGCA^H77C^/^H77C^	Knockin mouse	Normal		
LGMD2E	Sgcb^−/−^	Knockout mouse	Exon 2 disruption	AAV gene transfer (72)	Biochemical imp.
LGMD2F	BIO14.6	Spontaneous		AAV gene transfer (73)	Reverse morphology, function
				Tranilast, diltiazem (74)	Biochemical imp.
	TO-2	By breeding		AAV gene transfer (75)	Biochemical and functional
				HUCB i.myo. administration (76)	Short-term imp.
	Sgcd^−/−^	Knockdown	Exon 2 targeted replacement	Hematopoietic stem cells (77)	No imp.
				Myosphere-derived progenitors (78)	Improved heart function
				Myostatin blockade (79)	Early-stage imp.
				AAV gene transfer (80)	Heart but not muscle imp.
LGMD2G	TCap^−/−^	Knockout mouse			
LGMD2H	Trim32^−/−^	Knockout mouse			
	T32KI	Knockin mouse	Carries c.1459G>A, p. D487N mutation		
LGMD2J	TTN^+/c.43628insAT^	Knockin mouse (het)	Wild allele compensate mutated one		
	TTN^hom^	Knockin mouse (hom)	Lethal at E9.5		
	PEVK^−/−^	Knockout mouse	PEVK part of TTN highly phosphorylated		
	N2B^−/−^	Knockout mouse	Calcium-sensitive area of TTN		
	FIN^maj^	Knockout mouse	C-terminal area of TTN (homo. Het.)	FIN^maj^ (Het.) X capn3^−/−^mice (81)	Improve muscle, heart
LGMD2L	No model yet				
Dystroglycan					
	LARGE^myd^	Myodystrophy	LARGE gene mutated	LARGE gene transfer (82)	Improve structure and function
	LARGE^++/++^	Transgenic mouse			Late-onset loss of force
LGMD2I	FKRP-Neo^Tyr307Asn^	Knockin mouse	Lethal soon after birth		
	FKRP^Tyr307Asn^	Knockin mouse	Normal		
		FKRP−/−	Knockout mouse	Lethal at E12.5	
	FKRP^P448L/ P448L^	Knockin mouse	Structural anomalies reminiscent to human		
LGMD2K	POMT1^−/−^	Knockout	Lethal between E7.5 and E9.5		
LGMD2M	FKTN^+ETN^	Transgenic mouse	Retrotransposon insertion in FKTN gene		
LGMD2N	POMT2^−/−^	Knockout mouse	Lethal at E9.5		
LGMD2O	POMGnT1^−/−^	Knockout mouse	Muscle-eye-brain model		
LGMD2P	DGS654A	Transgenic mice	Inhibit dystroglycan cleavage		
	DAG1^T192M/ T192M^	Knockin mouse	Neuromuscular abnormalities		
LGMD2Q	No model yet				

LGMD, limb-girdle muscular dystrophies; C3, complement component C3; HUCB, human umbilical cord blood; imp., improvement; ref, reference; i.v., intravenous; i.a., intra arterial; i.myo., intramuscular; AAV, adeno-associated virus; DCM, dilated cardiomyopathy; HCM, hypertrophic cardiomyopathy.

LGMD, Limb-girdle muscular dystrophies; hom, homozygous; het, heterozygous.

**Table IV:** Relative % of different LGMD forms in different countries.

		LGMD						
		
Country (ref.)	No. of patients	2A	2B	2C-F	2I	2L	1B	1C
Italy (89)	228	37%	27%	23%	9%	2%	-	2%
Italy (90)	181	28.4%	18.7%	18.1%	6.4%	-	-	1.3%
Italy (91)	346	25.1%	11.2%	15%	4.3%	-	1.4%	1.4%
Spain (92)	-	80%	-	-	-	-	-	-
German (89)	124	-	-	-	16%	-	-	-
UK (86,94)	68	26.5%	5.9%	11.8%	19.1%	11.8%	8.8%	-
Norway (95)	326	-	-	-	27%	-	-	-
Denmark (96)	118	10.2%	1.7%	19%	32.2%	-	-	-
Finland (97)	101	-	-	-	-	25%	-	-
Australia (98)	76	8%	5%	2%	3%	-	1%	3%
USA (99)	226	12%	18%	15%	15%	-	-	1.5%
Mexico (87)	-	25%	40.6%	31.2%	-	-	-	3.1%
Turkey (100)	20	50%	5%	40%	-	-	-	-
Russia (101)	19	75%	-	-	-	-	-	-
Brazil (102)	-	32%	22%	32%	11%	-	-	-
China (In press)	68	17%	15%	3%	-	-	-	3%
Japan (103,104)	80	26%	Most	9%	-	-	-	-
India (105)	26	-	-	53.8%	-	-	-	-
India (106)	171	47%	-	-	-	-	-	
India (107)	30	21%	-	-	-	-	-	-

LGMD, limb-girdle muscular dystrophies; ref, reference.

**Table V:** LGMD: Common mutations with founder effects.

Type	Gene	Exon	Hot-spot mutations (exon no.)	Populations that express mutations (ref.)	Predicted phenotype
LGMD1A	*MYOT*	10	Exon 2	-	
LGMD1B	*LMNA*	12	-	-	
LGMD1C	*CAV3*	2	-	-	
LGMD1D	*DES*	9	-	-	
LGMD1E	*DNAJB6*	10	c.279C>G (E5)	Finland, Americans (13,14)	
LGMD2A	*CAPN3*	24	c.550delA (E4)	Russia, Czech, Turkey (40%), Italy, UK (101,155,156)	
			c.2362_2363delinsTCATCT (E22)	Spain (30%), Brazil (Hispanics) (92)	
			c.1469G4A, p.R490Q (E11)	Italy, Turkey (10%) (155,156)	
LGMD2B	*DYSF*	55	c.937+1G>A (E10)	Japan (104)	
			c.1566C>G, p.Y522X (E18)	Japan (104,157)	
			c.2997G>T, p.W999C (E28)	Japan, China, S. Korea (104)	Homozygous, mild
			c.3373delG, p.E1125KfsX1134 (E1)	Japan (104)	
			c.2494C>T, p.Q832X (E24)	S. Korea (158)	
			c.663+1G>C, splicing defect (E6)	S. Korea (158)	
			c.2372C>G, p.p791R (E24)	Canada (natives) (159)	
			c.2875C>T, p.R959W (E27)	Italy (159)	
			c.5713C>T, p.R1905X (E51)	Spain (159)	
			c.2779delG., p.A927LfsX21 (E26)	Caucasian Jewish population (159)	
			c.4872_4876delinsCCCC (E44)	Libyan Jewish population (159)	
LGMD2C	*SGCG*	8	-	-	
LGMD2D	*SGCA*	10	c.229C>T, p.R77C(E3)	Europe, Finland, Brazil (160)	Homozygous, mild
LGMD2E	*SGCB*	6	-	-	
LGMD2F	*SGCD*	9	-	-	
LGMD2G	*TCAP*	2	c.172C>T, p.Q53X (E2)	Brazil (141)	
LGMD2H	*TRIM32*	2	c.1459G>A, p.D487N (E2)	Hutterites (USA, Canada, Germany) (25,161)	
LGMD2I	*FKRP*	4	c.826C>A, p.L276I (E4)	Europe, American (90,95,96,162)	Homozygous, Becker like Heterozygous, Duchenne-like
LGMD2J	*TTN*	363	Mex6( 11-bp change)	Finland (148)	
LGMD2K	*POMT1*	20	c.598G>C, p.A200P (E7)	Turkey (27)	
LGMD2L	*ANO5*	22	c.191dupA, p.Asn64Lysfs*15 (E5)	Northern europeans (94)	Homogeneous phenotype
LGMD2M	*FKTN*	11	-	-	
LGMD2N	*POMT2*	21	-	-	
LGMD2O	*POMGnT1*	23	-	-	
LGMD2P	*DAG1*	6	-	-	
LGMD2Q	*PLEC*	33	c.1_9del, p.0(E2i)	Turkey (35)	

LGMD, limb-girdle muscular dystrophies.

**Table VI:** Update of therapeutic trials in humans.

Therapeutic option	Mechanism	LGMD form (ref.)	Comment
Gene therapy (rAAV)	I.M. Intact gene transfer	LGMD2D (85)	Application on larger and more functional muscle is required Intra-arterial delivery to whole-body muscles is warranted The study represents histological but not functional improvement
Rituximab (I.V) (monoclonal AB)	Against CD20-positive B cells 375 mg/m^2^/week (4 doses)	Miyoshi M (168)	Small number of patients, female responsiveness is requested Muscle adaptation to specific exercise should be considered Effect of treatment on quality of life is doubtful
Dantrolene (25 mg/day)	Ca^2+^ ion blocker in ER (ryanodine receptor binding)	Miyoshi M (169)	Query effect on weakness Hepatopathy side effect in up to 91%
Vitamin D3 (1/week for 1 year)	MEK/ERK pathway D3 receptor to DYSF promoter	DYSF carriers (170)	The study represents cohort of asymptomatic carriers
Deflazacort	Steroids (1 mg/kg/day)	DYSF-opathy (171)	Worsening of muscle strength
		LGMD2D (172)	Mildly symptomatic female patient
Prednisone	Steroids (1–2 mg/kg/day) (0.35 mg/kg/day)	LGMD2M (30)	Partial responsiveness, multiple fractures and susceptibility to infections
		LGMD2I (173)	Growth arrest, vertebral fractures and susceptibility to infection
Creatine MH	Helps to supply energy	Sarcoglycans (174)	Mild improvement (3%)
MYO-029	Neutralizing AB to myostatin	MD (167)	No improvements at end point
CoQ10 + lisinopril	Vitamin-like^+^ Ca^2+^ blocker	LGMD	Recruiting

LGMD, limb-girdle muscular dystrophies; AB, antibodies; MD, muscular dystrophies; I.M., intramuscular; I.V., intravenous; rAAV, recombinant adeno-associated virus.
